# Lithium and the Interplay Between Telomeres and Mitochondria in Bipolar Disorder

**DOI:** 10.3389/fpsyt.2020.586083

**Published:** 2020-09-29

**Authors:** Martin Lundberg, Vincent Millischer, Lena Backlund, Lina Martinsson, Peter Stenvinkel, Carl M. Sellgren, Catharina Lavebratt, Martin Schalling

**Affiliations:** ^1^Department of Molecular Medicine and Surgery, Karolinska Institutet, Stockholm, Sweden; ^2^Center for Molecular Medicine, Karolinska University Hospital, Stockholm, Sweden; ^3^Centre for Psychiatry Research, Department of Clinical Neuroscience, Karolinska Institutet & Stockholm Healthcare Services, Region Stockholm, Stockholm, Sweden; ^4^Department of Clinical Science, Intervention and Technology, Karolinska Institutet, Stockholm, Sweden; ^5^Department of Physiology and Pharmacology, Karolinska Institutet, Stockholm, Sweden

**Keywords:** lithium, bipolar disorder, mitochondria, oxidative stress, telomere, telomerase, telomerase reverse transcriptase, GSK3

## Abstract

Bipolar disorder is a severe psychiatric disorder which affects more than 1% of the world’s population and is a leading cause of disability among young people. For the past 50 years, lithium has been the drug of choice for maintenance treatment of bipolar disorder due to its potent ability to prevent both manic and depressive episodes as well as suicide. However, though lithium has been associated with a multitude of effects within different cellular pathways and biological systems, its specific mechanism of action in stabilizing mood remains largely elusive. Mitochondrial dysfunction and telomere shortening have been implicated in both the pathophysiology of bipolar disorder and as targets of lithium treatment. Interestingly, it has in recent years become clear that these phenomena are intimately linked, partly through reactive oxygen species signaling and the subcellular translocation and non-canonical actions of telomerase reverse transcriptase. In this review, we integrate the current understanding of mitochondrial dysfunction, oxidative stress and telomere shortening in bipolar disorder with documented effects of lithium. Moreover, we propose that lithium’s mechanism of action is intimately connected with the interdependent regulation of mitochondrial bioenergetics and telomere maintenance.

## Introduction

Bipolar disorder (BD) is a chronic psychiatric disorder characterized by recurrent episodes of mania or hypomania and depression (1). It affects >1% of the world’s population (2) and is a major cause of disability, especially in young people (3). The risk of suicide in BD is up to 20 times higher than in the general population, with approximately one third to half of patients attempting suicide at least once during their lifetime and roughly 15–20% of attempts being completed (4). BD is also associated with a high degree of psychiatric comorbidities, including anxiety disorders, attention-deficit/hyperactive disorders, personality disorders, and substance use disorders (5); as well as medical comorbidities, including cardiovascular disorder, diabetes, obesity, and dementia (6, 7). Mortality rate is increased in BD, particularly due to the high rate of suicide and cardiovascular disorders (8). Furthermore, a subset of BD patients seems to present with a progressive course of illness, characterized by worsening of symptoms and increasing treatment resistance over time, as well as functional and neurocognitive impairments that remain during periods of remission (9). Accurate diagnosis of BD in early stages of the disorder is challenging and there are thus far no clinically useful biomarkers to aid in diagnostics (10). This often results in a delay of several years from onset of symptoms to diagnosis and adequate treatment with mood-stabilizers (11). This delay poses a significant problem, as duration of untreated illness is an important prognostic factor in BD and longer duration of untreated illness has been associated with higher frequency of suicide attempts (12).

The etiology and pathophysiology of BD is complex and far from understood (13). BD is one of the most heritable psychiatric disorders, with an estimated heritability of 60–85%, though both genetic and environmental factors influence risk (14–16). Thus far, 30 genome-wide significant loci have been identified in BD, containing genes encoding ion channels, neurotransmitter transporters and synaptic components (17). The pathophysiology of BD is characterized by multiple biological findings, including disturbed brain development, neuroplasticity and chronobiology; defects in apoptotic, immune-inflammatory, neurotransmitter, neurotrophic, and calcium signaling pathways; oxidative and nitrosative stress; mitochondrial dysfunction; and endoplasmic reticulum stress, recently summarized by Sigitova et al. (13). Progressive neuroanatomical changes; including reduced cortical thickness in frontal, medial parietal and occipital regions (18), decreased hippocampal volume (19) and enlarged lateral ventricles (20); associated with functional and neurocognitive impairments, have been observed in a subset of BD patients and is encompassed by the term neuroprogression (9). Moreover, altered levels of several markers of biological aging have been reported in BD, including decreased mitochondrial DNA (mtDNA) copy number as well as shorter telomere length (TL), which may indicate accelerated aging (21). In an attempt to integrate the multitude of findings, several hypotheses regarding BD pathophysiology have been proposed. Many of these converge on a model of disrupted brain energy metabolism in which mitochondrial dysfunction results in calcium dysregulation and altered neuronal excitability, as well as oxidative stress, apoptosis, and neuroinflammation (22–24). Furthermore, accumulated tissue damage as a result of repeated exposure to cellular stressors and overactivation of allostatic systems has been suggested as a potential cause of neuroprogression and accelerated aging in BD (9, 25).

Lithium is commonly used as the first-line long-term treatment option in BD due to its potent effect on preventing both manic and depressive episodes (26) and on reducing the risk of suicide (27). In addition, lithium treatment has been associated with lower cardiovascular and all-cause mortality (28–30). Interestingly, lithium has in recent years also been suggested as a potential pharmacotherapy in several neurodegenerative disorders, including Parkinson’s disease, Alzheimer’s disease and amyotrophic lateral sclerosis, due to its neuroprotective properties (31, 32). However, the benefits of lithium are restricted by several adverse effects and a narrow therapeutic index (33). Moreover, while approximately a third of BD patients responds very well to lithium, one third does not respond at all, and there are no clinically useful biomarkers to predict lithium response (34).

Lithium’s mechanism of action is also complex, with a multitude of effects within different cellular pathways and biological systems (35). These include the inhibition of inositol monophosphatase (IMPA) and glycogen synthase kinase 3-beta (GSK3-beta) as well as various effects on neurotrophic factors, neurotransmitters, mitochondrial respiration and oxidative metabolism, apoptosis, neuronal structures and glia, second messenger systems, and biological systems such as circadian rhythm and the hypothalamic-pituitary-adrenal axis, recently summarized by Malhi and Outhred (35). Moreover, recent studies have indicated that long-term lithium treatment in BD patients may counteract telomere shortening and potentially increase TL (25). Although lithium has been widely studied over the years, its specific therapeutic action in BD is still largely unknown (35).

Though historically studied primarily in separate, it has in recent years become clear that mitochondria and telomeres are intimately linked. On the one hand, mitochondrial dysfunction leads to an increased production of reactive oxygen species (ROS) which can cause telomere damage. Conversely, telomere shortening can lead to reprogramming of mitochondrial biosynthesis and dysfunctional mitochondria. Interestingly, telomerase reverse transcriptase (TERT) has been identified as a central enzyme in ameliorating this vicious loop through both telomere-dependent and -independent actions (36).

In this narrative review, we outline the current understanding of mitochondrial dysfunction, oxidative stress, and telomere shortening in BD and the effects of lithium on counteracting these cellular lesions. Moreover, we discuss the interdependent regulation of mitochondrial bioenergetics and telomere maintenance through ROS signaling and the subcellular translocation and actions of TERT. Finally, in an attempt to integrate the various cellular effects of lithium, we propose a novel hypothesis of lithium’s mechanism of action which includes the regulation of TERT and its subsequent mitochondrial and telomeric effects. We hope that this may open up new avenues of lithium research and aid in the search for biomarkers and new pharmacological targets in BD.

## Mitochondrial Bioenergetics

### Mitochondrial Biology

Mitochondria are semiautonomous, self-reproducing organelles, present in the cytoplasm of most eukaryotic cells and responsible for producing the majority of the cell’s ATP through oxidative phosphorylation (37). Mitochondria evolved from cyanobacteria that were engulfed by proto eukaryotes more than 1.5 billion years ago and have retained many of the characteristics of their bacterial ancestors, including reproduction through a fission process and a bacterial-like biosynthetic machinery. Although mitochondria have retained their own genomes, many genes required for the execution of their specialized functions were relocated to the nucleus during the development of their symbiotic relationship with eukaryotic cells (38).

Mitochondria are very adaptable and can adjust their location, shape, and number to suit the needs of different types of cells, while retaining the same basic internal structure (37). They are organized into four separate compartments: two membranes, one inside the other, forming a large internal space known as the matrix and a narrow intermembrane space. The mitochondrial matrix contains hundreds of enzymes, including those required for the oxidation of pyruvate and fatty acids and for the citric acid cycle. The inner membrane, which is folded into cristae, contains the proteins required for oxidative phosphorylation as well as transport proteins. The outer membrane contains large, channel-forming proteins, called porins, which allow small molecules and ions to diffuse freely. Finally, the intermembrane space contains proteins released during apoptosis as well as enzymes that use ATP to phosphorylate other nucleotides (37).

Cells produce the majority of their energy through the two-stage process of oxidative phosphorylation in the inner membranes of mitochondria. In the first stage, high-energy electrons derived from the oxidation of nutrients are transferred along four electron transport chain (ETC) complexes, labeled I–IV, to their final acceptor, oxygen, to form water. With the exception of complex II, each electron transfer releases energy used to pump protons across the membrane, generating an electrochemical proton gradient. In the second stage, protons flow back down their electrochemical gradient through the membrane embedded protein complex ATP synthase (sometimes denoted complex V), which catalyzes the synthesis of ATP from ADP and inorganic phosphate. Without oxidative phosphorylation, cells would have to rely on the relatively inefficient process of glycolysis which, comparatively, only produces a fraction of the ATP from each molecule of glucose (37).

In addition to supplying the majority of the cell’s energy, mitochondria play crucial roles in pathways of cellular resilience and death, generation of oxidative and nitrosative stress, regulation of intracellular calcium signaling, and in regulating inflammation (39).

### Mitochondrial Dysfunction in Bipolar Disorder

The human brain has a high energy demand, the majority of which is generated by mitochondria in the form of ATP, rendering it especially susceptible to mitochondrial lesions (40). Mitochondrial dysfunction and abnormal brain energy metabolism have been implicated as key aspects of the pathophysiology of BD through multiple lines of evidence from imaging, post-mortem, gene expression, and cellular studies (24).

Magnetic resonance spectroscopy studies have identified decreased levels of creatine and phosphocreatine as well as increased levels of lactate and decreased pH in the brains of BD patients, indicating impaired oxidative phosphorylation and a shift toward glycolysis for energy production (41, 42). Moreover, several studies have found decreased cerebral concentrations of N-acetyl aspartate (NAA) in BD patients (41–44). NAA, which is the second most abundant amino acid in the central nervous system after glutamate, is synthesized by mitochondria, and decreased levels of NAA could indicate impaired mitochondrial energy production (41, 45, 46). Mitochondrial morphological abnormalities have also been observed in BD patients, including smaller mitochondria in post-mortem brain tissue and patterns of mitochondrial clumping and marginalization in peripheral cells (47). Mixed results regarding mtDNA copy number, corresponding to mitochondrial function and biogenesis, have been reported in BD. While several studies reported decreased mtDNA copy number in BD (48–50), some have found no difference between patients and controls (51, 52). Moreover, one study found mtDNA copy number to be increased in BD patients (53). Interestingly, the expression of polymerase gamma (POLG), the only enzyme known to replicate mtDNA, has been shown to be increased in BD patients (54). A compensatory upregulation of POLG in order to counteract mtDNA damage has been suggested to explain this finding (54).

Several post-mortem studies have found both downregulation and upregulation of mitochondria-related genes; particularly those encoding complexes of the ETC (55–59). Moreover, decreased complex I activity has been reported in the prefrontal cortex of BD patients (55). In a landmark study, Mertens et al. (60) investigated the cellular phenotypes of hippocampal dentate gyrus-like neurons derived from induced pluripotent stem cells (iPSCs) from patients with BD. Guided by RNA sequencing expression profiling, they detected mitochondrial abnormalities in young neurons from BD patients in terms of increased expression of multiple mitochondrial genes, increased mitochondrial membrane potential, and smaller mitochondria. In addition, using patch-clamp recordings and somatic calcium imaging, they detected hyperactive action potential firing. The authors hypothesized that the smaller size of mitochondria and higher mitochondrial membrane potential may result in faster mitochondrial transport and increased energy production which, in turn, could explain the increased action potential firing, corresponding to the manic state of BD (60). Finally, in patients with mitochondrial diseases, the prevalence of BD is nearly 20 times higher than in the general population (61–63).

### Lithium’s Effect on Improving Mitochondrial Function

Multiple lines of evidence from clinical and animal studies as well as from human cellular models have implicated mitochondrial energy metabolism as a target of lithium compounds. Using spectrophotometry, lithium treatment has been shown to increase ETC complex I, II, and III activity in the frontal cortex of post-mortem brains from BD patients (64). Moreover, in a study by de Sousa et al. (65), lithium treatment was shown to increase ETC complex I activity in leukocytes from BD patients in a plasma lithium level-dependent manner (65).

D-amphetamine (d-AMPH)-induced hyperactivity in rats has been proposed as an animal model of mania. In this model, repeated injections of d-AMPH has been shown to cause inhibition of ETC complexes I, II, III, and IV in the hippocampus, striatum and prefrontal cortex. Conversely, pre-treatment of these rats with lithium has been shown to attenuate hyperactive behavior and prevent complex I and II inhibition in the striatum and prefrontal cortex, complex III inhibition in the striatum and hippocampus, and complex IV inhibition in the striatum (66). Another suggested animal model of mania is N-methyl-d-aspartate (NMDA)-induced hyperactivity in rats. Repeated NMDA injection has been shown to decrease brain levels of mitochondrial complex I and III and increase levels of lipid peroxidation products 8-isoprostane and 4-hydroxynonenal while pre-treatment with lithium prevented lipid peroxidation (67). Furthermore, treatment of rat cortical primary neurons with the ETC complex I inhibitor rotenone causes increased DNA methylation and hydroxymethylation levels and increased cell death and apoptosis, in addition to decreased complex I activity and ATP production. Pre-treatment of these cells with lithium counteracted these effects, demonstrating a protective role of lithium on mitochondria (68). Moreover, rotenone has been shown to counteract lithium’s mood-stabilizing effect on mice behavior, suggesting a central role of mitochondrial bioenergetics in the mood-stabilizing action of lithium (69).

In a study by Corena-McLeod et al. (70), chronic treatment with lithium resulted in high levels of phosphorylation of cytoskeletal and mitochondrial proteins in synaptoneurosomal preparations from rat prefrontal cortex. The authors also demonstrated that lithium treatment increased the phosphorylation of the protein UQCRC1, a component of ETC complex III. They hypothesized that lithium modulates both mitochondrial migration and energy production (70). Lithium has also been found to upregulate the expression of several mitochondria-related genes, including *COX5A*, *NDUFS7*, *NDUFAB1*, in mouse frontal cortex (71). Furthermore, the hyperexcitable phenotype of hippocampal dentate gyrus-like neurons derived from iPSCs from BD patients described by Mertens et al. (60) was selectively reversed by lithium treatment only in neurons derived from patients who also responded to lithium treatment (60).

However, deleterious effects of lithium on mitochondrial function have also been observed in some animal studies. Lithium treatment of cardiomyocytes isolated from the heart of the Wistar rat has been shown to induce a concentration- and time-dependent rise in mitochondrial ROS formation, inhibition of ETC complex II, mitochondrial membrane potential collapse, mitochondrial swelling, and cytochrome c release. Moreover, lithium was found to induce caspase 3 activation through the mitochondrial pathway, decrease ATP/ADP ratio, and increase lipid peroxidation (72). In another study, lithium treatment of isolated pig brain mitochondria was shown to inhibit ETC complex I activity (73). The difference in results in terms of lithium’s effect on mitochondrial function may be explained by methodological differences, both in terms of tissue type and of lithium concentration used to treat cells. This is highlighted in a study by Pietruczuk et al. (74), demonstrating that therapeutic concentrations of lithium stabilized mitochondrial membrane polarization and decreased apoptosis, while higher concentrations caused mitochondrial depolarization and induced apoptosis in cultured lymphoid cells (74).

## Oxidative Stress

### Redox Homeostasis

Redox homeostasis describes the balance between intracellular oxidants and antioxidants. A disruption of this balance, either due to increased ROS production or decreased levels of antioxidants, results in oxidative stress (75). While the high energy output of oxidative phosphorylation in mitochondria is a prerequisite for complex multicellular animal life, it is also the main source of endogenous oxidative stress (37, 76). 1–3% of all electrons transferred through the ETC leaks to generate ROS, particularly through complex I. If ROS are not detoxified by antioxidants, they can cause toxic effects through oxidization of DNA, proteins and lipids (76). Generation of ROS in mitochondria can be increased either as a consequence of diminished electron transport when ATP production exceeds the energy demand of the cell, or when specific respiratory chain complexes become impaired or uncoupled. Accumulated oxidative damage, particularly in terms of telomere shortening and mtDNA damage, are believed to be central in cellular and organismal aging and plays a key role in the development of multiple diseases including cardiovascular and neurodegenerative disorders, diabetes and cancer (76). On the other hand, ROS also serve as signaling molecules, regulating cellular proliferation and survival through several pathways (75).

### Oxidative Stress in Bipolar Disorder

The human brain is highly susceptible to oxidative stress due to its lipid-rich content and high oxygen consumption, and dysregulated redox homeostasis is frequently reported in both neurodegenerative and neuropsychiatric disorders (77). This is true also for BD, in which increased levels of multiple oxidative stress markers have been reported.

These include (1) increased levels of 8-oxo-2´-deoxyguanosine, a marker of oxidative DNA damage (78), (2) increased levels of 8-oxo-7,8-dihydroguanosine, a marker of oxidative RNA damage (78), (3) increased levels of lipid hydroperoxide (79) and thiobarbituric acid reactive substances (TBARS) (80), markers of lipid peroxidation, and (4) increased levels of protein carbonyl, a marker of oxidative protein damage (80). Alterations in levels of antioxidant enzymes have also been reported in BD. Reduced levels of glutathione peroxidase have been noted (81) while increased levels of glutathione transferase and glutathione reductase have been reported in late stage BD (82).

### Lithium’s Effect on Decreasing Oxidative Stress

Several studies in both humans, animals, and cultured cells have demonstrated antioxidative properties of lithium. In manic BD patients, acute treatment with lithium has been shown to decrease the superoxide dismutase (SOD)/catalase ratio as well as to decrease levels of TBARS (83). These results were replicated in a subsequent study which showed a decrease in levels of SOD and TBARS in BD patients after 6 weeks of lithium treatment. Moreover, lithium responders were shown to have significantly lower levels of TBARS than non-responders (52). In healthy volunteers, 2–4 weeks of lithium treatment in therapeutic doses decreased levels of SOD, demonstrating an antioxidant effect of lithium, independent of disease status (84).

In terms of animal studies, lithium has been shown to prevent and reverse d-AMPH and methamphetamine induced oxidative protein and lipid damage in the brain of rats, in addition to preventing and reversing hyperactivity (85–88). Additionally, chronic treatment with lithium at therapeutic concentrations was reported to significantly inhibit glutamate-induced increase of intracellular free Ca2+ concentration, lipid peroxidation, protein oxidation, DNA fragmentation, and cell death in primary rat cerebral cortical cells (89).

In cultured human neurons, lithium treatment has been found to increase resistance to oxidative stress and cellular growth rate, enhance glucose consumption and glycolytic activity and increase levels of the antioxidant glutathione and the anti-apoptotic protein B-cell lymphoma 2 (90).

## Telomere Maintenance

### Telomere Biology

Telomeres are nucleoprotein structures at the ends of chromosomes consisting of non-coding hexameric tandem repeats of *TTAGGG* in association with several specialized proteins, including the protective shelterin complex. They prevent the end of linear chromosomes from being recognized as broken ends, averting processes such as DNA end joining, DNA recombination, and DNA repair, that would otherwise cause chromosomal instability (91). However, as DNA polymerase can only proceed in a 5´to 3´direction and requires a primer with a 3´ end, it is unable to copy the last part of the lagging strand during replication, resulting in a progressive shortening of telomeres at every cellular division (91). This phenomenon, known as the “end-replication problem” (92, 93), is resolved in eukaryotes through the enzyme telomerase, which can add telomeric repeats to the ends of chromosomes and thus compensate for telomere shortening (94). However, as the expression and activity of telomerase is limited in most human cell types, telomeres shorten throughout the lifespan until a critical limit, known as the “Hayflick limit”, is reached (95), which triggers a form of DNA damage signaling, altering transcriptional profiles and causing the cell to become senescent (96). TL is thus an important biological marker of cellular aging and accelerated telomere shortening is associated with several common, and often comorbid, age-related disorders, as well as increased mortality (96).

Telomerase consists of two main components: telomerase RNA component (TERC), an RNA template used for the synthesis of telomeres; and TERT, a catalytic subunit; together with associated proteins (97). In addition to contributing to the canonical telomere-lengthening effect of telomerase, TERT and TERC have been found to perform additional, non-canonical, function in the nucleus, cytosol and mitochondria: influencing gene expression, signaling pathways, mitochondrial function, and resistance to cellular stress and degradation (36). *TERT* is expressed together with *TERC* in cells with high replicative capacity such as stem cells, germ cells and tumors, where telomerase activity is necessary for prolonged survival. However, in recent years, it has become clear that *TERT* is also expressed to a lesser extent in tissues with low replicative potential such as vasculature (98, 99), the hearth (100, 101), and the brain (102, 103).

### Telomere Shortening in Bipolar Disorder

Whether BD is associated with accelerated telomere shortening or not has been somewhat controversial. While several studies have reported shorter telomeres in BD patients (81, 104–109), both in early and late stages of the disease (104), others have found no association between BD and TL (53, 110–112). In one study, TL was even found to be increased in BD patients compared to healthy controls (113). To address this uncertainty, a meta-analysis of 570 patients and 551 controls across ten different studies was recently performed which found that telomeres were significantly shorter in BD patients compared to healthy controls, regardless of mood state or method used to measure telomeres (114). Interestingly, one study found shorter TL in both BD patients as well as their unaffected siblings. The authors concluded that shorter telomeres may constitute a genetic risk of developing BD (81). However, this may also be explained by the inherent heritability of TL (115).

The variety of results from individual studies may be explained by differences in terms of important clinical, demographic, and pharmacological factors that may influence TL in BD. In terms of clinical characteristics, two studies have found associations between number of depressive episodes and shorter TL in BD (106, 113), although not replicated in a subsequent study (107). BD has been associated with obesity and smoking, both of which has been shown to correlate negatively with TL (116–120). Moreover, lithium treatment has been associated with longer telomeres in BD patients (113, 121). As the previously mentioned studies controlled for these potential confounders to varying degrees and none controlled for all, the results become difficult to interpret. Thus, although telomere shortening in BD is frequently reported, additional studies are needed to clarify this relationship.

### Lithium’s Effect on Increasing Telomere Length

Several clinical studies indicate that lithium may attenuate telomere shortening and possibly increase TL. The initial finding came from a study by our group, in which we found that lithium-treated BD patients overall, as well as those on lithium monotherapy had 35% longer telomeres than controls (113). Moreover, TL was positively correlated with duration of lithium treatment in patients treated for more than 30 months and lithium responders had longer TL than non-responders (113). Subsequently, several studies have replicated this initial finding. Squassina et al. (121) similarly found a positive correlation between duration of lithium treatment and leukocyte TL in patients treated for more than two years (121). Powell et al. (122) reported longer TL in buccal smears from lithium-treated BD patients compared to patients not on lithium treatment while Coutts et al. (123) found an association between chronic lithium treatment and longer whole blood TL in BD patients (122, 123). Most recently, Pisanu et al. (124) reported that BD patients with a history of lithium treatment had longer leukocyte TL compared to those who never had been treated with lithium and similar TL compared to controls (124). In the only prospective clinical study on lithium’s effect of TL thus far, Köse Çinar (125) found that manic BD patients had shorter TL than healthy controls and that TL was significantly increased at remission after treatment with lithium and antipsychotics (125).

The effect of lithium treatment on TL has also been studied in several animal models. In a study by our group, we found that six weeks of lithium treatment in a rat model of depression, The Flinders Sensitive Line, significantly increased TL (126). Subsequently, using a triple-transgenic mouse model of Alzheimer’s Disease (3xTg-AD), Cardillo et al. (127) determined the effect of chronic lithium treatment on TL in different regions of the mouse brain. They found that chronic lithium treatment was associated with longer telomeres in the hippocampus and parietal cortex and that the magnitude of the telomere-lengthening effect of lithium was dependent on lithium concentrations and characteristics of the tissue (127).

## Dual Roles of Telomerase Reverse Transcriptase

### Interactions Between Mitochondrial Bioenergetics and Telomere Maintenance

Mitochondrial dysfunction, oxidative stress, and telomere shortening are intimately linked (128). mtDNA damage has been shown to accumulate during the human lifespan and is believed to be a result of replicative stress generated by mtDNA replication errors and oxidative stress generated by the inherent leakiness of the ETC (129). As a result of this genetic deterioration, mitochondrial respiration becomes more and more inefficient, resulting in a progressive increase in ROS production. The guanin-rich nature of telomeric repeats makes telomeres particularly susceptible to oxidative stress and, when exposed to ROS, they are prone to develop stretches of 8-oxoguanine (8-oxoG) which are difficult to repair (130). These lesions can result in single- and double-stranded DNA breaks and generate replicative stress, which ultimately leads to telomere shortening (131, 132).

Telomeric DNA (telDNA) damage and telomere shortening, on the other hand, can lead to mitochondrial dysfunction and reprogramming of mitochondrial biosynthesis through several different pathways (36). The negative effect short telomeres have on mitochondrial function is believed to be caused by an increase in DNA damage responses (DDRs) (133). Overexpression of the dominant-negative telomere-binding protein telomeric repeat-binding factor 2, which induces purely telomere-dependent senescence, has been shown to activate p21, which in turn results in mitochondrial dysfunction and increased production of ROS (133). Moreover, telomere shortening has been shown to activate p53 which binds to proliferator-activated receptor gamma, coactivator 1 alpha and beta (PGC-1alpha and PGC-1beta) promoters, suppressing their expression, which downregulates mitochondrial biosynthesis (134). DDRs have also been shown to inhibit PGC-1beta through the ATM, Akt, and mTORC1 phosphorylation cascade, decreasing mitochondrial content (135).

To counteract the progressive decline in mitochondrial function and telomere shortening caused by oxidative stress, cells have developed defense mechanisms to repair and prevent oxidative damage of telDNA and mtDNA (128). In response to telDNA damage by ROS, 8-oxoguanine DNA glycolylase (OGG1) is recruited to telomeres to repair stretches of 8-oxoG (136). Moreover, peroxiredoxin 1 localizes at telomeres in response to oxidative stress and recruits 7,8-dihydro-8-oxoguanine triphosphatase which is involved in deoxyguanosine triphosphate clearance (137, 138). mtDNA share many structural features with telDNA that similarly makes it susceptible to ROS damage and prone to develop 8-oxoG (128). In mitochondria, these lesions are also repaired by OGG1 as well as by base excision repair (139, 140). In order to combat oxidative stress at its source, specialized mitochondrial proteins such as TERT, TERC, and TRF-interacting protein 2 shuttle between nucleus and mitochondria and by doing so, modulates mitochondrial metabolism and ROS generation in a feedback manner (141–143). Of these proteins, TERT is believed to have a central role in improving mitochondrial function and decreasing oxidative stress (144).

### Non-Canonical Functions of Telomerase Reverse Transcriptase

**Figure 1:**
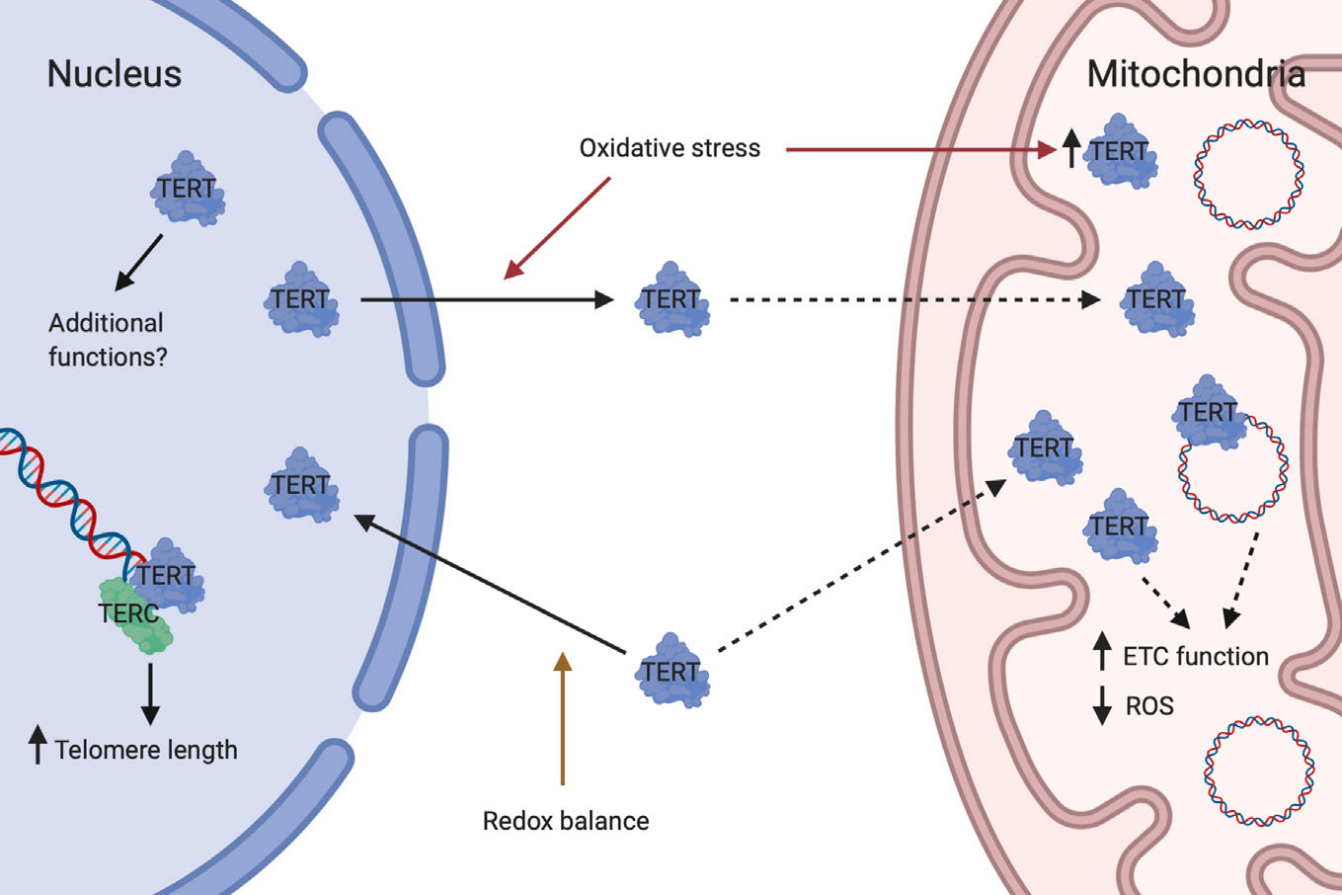
Dual roles of telomerase reverse transcriptase. Under conditions of redox balance, TERT is mainly transported to the nucleus where it complexes with TERC to form telomerase which lengthen telomeres. Nuclear TERT may also have additional functions. In response to oxidative stress, TERT is exported from the nucleus and mitochondrial TERT is concomitantly increased. Whether this is due to direct shuttling from the nucleus to mitochondria or a result of increased import of newly synthesized TERT into mitochondria is currently unknown. In mitochondria, TERT binds to and protects mtDNA and may perform additional actions. Mitochondrial TERT is associated with improved ETC function and reduced ROS generation. Created with Biorender.com.

Although historically studied mainly for its telomere-lengthening effect as part of the telomerase complex, it has in recent years become evident that TERT performs several additional, non-canonical functions and seems to be particularly important in the crosstalk between nucleus and mitochondria (**Figure 1**) (36). Important non-canonical functions of TERT include stabilization of the ETC, maintenance of cellular redox homeostasis and protection against apoptosis (144). Intriguingly, TERT and ROS are interdependent, with TERT controlling ROS levels in the cytosol and mitochondria and itself being regulated by changes in redox balance (144).

The transcriptional regulation of *TERT* is very complex, varies between different cell types and is still not completely understood. The human *TERT* promoter contains binding sites for multiple transcription factors including MYC, SP1, ER, AP1, ETS, HIFs, as well as upstream stimulatory factors (145). MYC is a central regulator of *TERT* transcription which can increase *TERT* expression independently of cellular proliferation state and *de novo* protein synthesis (146).

Subcellular localization of TERT seems to be primarily regulated by changes in intracellular redox homeostasis. During physiological conditions, approximately 20–30% of TERT is located outside of the nucleus and partially within mitochondria. However, during oxidative stress this relationship is reversed with approximately 80–90% of TERT becoming localized to mitochondria (141, 147). A nuclear import signal has been identified at amino acid residues 220–240 of TERT (148). In a balanced redox state, protein kinase B (Akt) facilitates nuclear import of TERT through phosphorylation at serine 227 (148). Upon short-term oxidative stress, TERT is exported from the nucleus with a concomitant increase in the mitochondria (98, 147). Currently it is unclear whether TERT is shuttled directly from nucleus to mitochondria or if newly synthesized TERT is imported into mitochondria. Nuclear export is facilitated by the phosphorylation of TERT at tyrosine 707 by Src kinase (98, 147). This step can be inhibited by the tyrosine phosphatase Shp-2, which dephosphorylates TERT (149). A nuclear export signal motif at the C-terminus of TERT has been identified, which interacts with the nuclear export receptor CRM1/exportin and facilitates translocation of TERT to the cytoplasm (150). The export requires an active nuclear Ran GTPase (151) and can be inhibited by 14-3-3 proteins which binds to TERT (150). Import of TERT into the mitochondrial matrix is dependent on a mitochondrial localization sequence at the N-terminus of TERT (141, 152). An active mitochondrial import mechanism has been suggested, based on observations of interaction of TERT with TOM20 and TOM40 at the mitochondrial outer membrane and with TIM23 at the inner membrane (141). Sustained oxidative stress finally leads to a reduction in mitochondrial TERT levels, a process which also appears to be mediated by Src kinase (153).

Mammalian target of rapamycin (mTOR) has also recently been implicated in the regulation of TERT localization. In a study by Miwa et al. (154), using a mouse model of Alzheimer’s Disease, inhibition of mTOR signaling, either through dietary restriction or treatment with the mTOR inhibitor rapamycin, increased the localization of TERT at mitochondria in the brain, which in turn was associated with decreased production of ROS (154).

Within the mitochondrial matrix, TERT binds to mtDNA at *ND1* and *ND2*, encoding two of the seven subunits of ETC complex I and has been shown to protect mtDNA against oxidative stress (141). TERT has also been shown to interact with the RNA component of mtRNA processing endoribonuclease (RMRP) to form a distinct ribonucleoprotein complex with RNA-dependent RNA polymerase activity that produces double-stranded RNAs which can be processed into small interfering RNAs (155). Moreover, TERT has been shown to interact with mitochondrial transfer RNAs (tRNAs) and 5.8S rRNA, in addition to RMRP. It has also been demonstrated that, in a cell-free extract, TERT functions as a TERC-independent reverse transcriptase, using mitochondrial tRNAs as templates (156).

Mitochondrial localization of TERT has been associated with an increase in ETC complex I and IV activities (157), increased mitochondrial membrane potential (138), decreased mitochondrial ROS production (98, 141), increased expression of the antioxidant manganese superoxide dismutase (MnSOD) (158), decreased nuclear and mitochondrial DNA damage, increases in mtDNA copy number, decreased cytochrome c release and apoptosis (151, 158–160), and overall improved survivability of cells (141, 147, 161–164).

The various mitochondrial effects of TERT has been suggested to stem from its direct and indirect interactions with mtDNA. In a recent publication, Rosen et al. (144), hypothesized that damage to mtDNA may compromise the assembly of respiratory chain complexes leading to increased ROS production. In this context, mitochondrial TERT would bind to and protect mtDNA, thus maintaining cellular redox homeostasis (144). Moreover, in a review by Billard and Poncet (128), mitochondrial TERT was hypothesized to regulate the concentration of mitochondria-encoded tRNA and influence the replication and transcription of mtDNA, thus controlling a feedback loop regulating mitochondrial bioenergetics and ROS production (128).

## Potential Role of Lithium in the Regulation of Telomerase Reverse Transcriptase

As outlined in this review, several similarities exist between the effects of lithium and TERT on mitochondrial bioenergetics, redox homeostasis, and telomere maintenance, including increased expression and activity of ETC complexes, decreased ROS generation and oxidative stress, increased TL, and increased overall survivability of cells. Based on this overlap we hypothesize that an upregulation of *TERT* expression could constitute part of lithium’s mechanism of action. Nuclear localization of TERT and its subsequent complexing with TERC to form telomerase provides a potential explanation for lithium’s effect, not only on attenuating telomere shortening, but also on seemingly lengthening telomeres. Mitochondrial localization of TERT and its subsequent interaction with mtDNA, on the other hand, provides a possible explanation for lithium’s effect on improving mitochondrial respiration and decreasing ROS production. Moreover, a reduction in ROS generation could also contribute to lithium’s effect on attenuating telomere shortening.

Corroborating this hypothesis are two studies from our group in which we investigated the associations between lithium treatment and *TERT* expression. In the initial study, we found that six weeks of lithium treatment increased hippocampal *TERT* expression in a rat model of depression (126). In the second study we found that *TERT* expression was significantly increased in lithium treated BD patients as well as a positive correlation between duration of lithium treatment and *TERT* expression (112).

Although lithium’s mechanism of action remains incompletely understood, inhibition of IMPA and GSK-3, are considered to be central to its mood-stabilizing and neuroprotective effects (165, 166). Inhibition of GSK-3 impacts several different signaling pathways implicated in neuronal function that also regulate *TERT* expression including the Wnt/beta-catenin pathway and the nuclear factor (erythroid-derived 2)-like 2 (Nrf2) pathway (167, 168). Moreover, lithium has been shown to increase CREB-dependent transcription of brain-derived neurotrophic factor (BDNF), which also upregulates TERT (166, 169).

Through the Wnt/beta-catenin pathway, inhibition of GSK-3 by lithium prevents the degradation of beta-catenin and enables its translocation to the nucleus where it binds to the T-cell factor/lymphoid factor (TCF/LEF) family of transcription factors (170). Subsequent transcription of Wnt/beta-catenin target genes leads to regulation of diverse processes critical for the central nervous system such as synapse plasticity and neurogenesis (170). One of the genes targeted by beta-catenin is *c-Myc*, which in turn activates *TERT* transcription (171–173). Nrf2, on the other hand, has been implicated as an important mediator of antioxidant effects of lithium and GSK-3 inhibition (174). Nrf2 responds to oxidative stress by increasing expression of an array of antioxidant response elements-containing genes to counteract oxidative damage (175). Nrf2 has recently also been shown to upregulate *TERT* expression *via* MYC and SP1 (176, 177). Moreover, lithium upregulates basal adenylate cyclase activity, and thereby cyclic AMP and protein kinase A, which results in CREB-dependent transcription of *BDNF* (178). BDNF is believed to be a key mediator of lithium-mediated neuroprotection (179) and has also been shown to induce the expression of *c-Myc via* the MAPK/PI3K pathway which, in turn, activates *TERT* expression (180).

As improvement of energy regulation and reduction of oxidative stress is believed to be important to lithium’s mood-stabilizing and neuroprotective effects (35), we further hypothesize that the propensity and ability of cells to transport TERT to mitochondria may be of importance for lithium response in BD.

Through the inhibition of GSK-3-beta, lithium activates Akt (181). Since Akt facilitates the nuclear import of TERT, lithium may act to direct TERT to the nucleus under physiological conditions when Akt is not inactivated by oxidative stress. Moreover, inhibition of mTOR, a central mediator of autophagy, has been shown to increase localization of TERT at mitochondria and reduce the production of ROS (154). Lithium has been shown to induce autophagy through mTOR-independent mechanisms *via* inhibition of IMPA, leading to depletion of free inositol and reduced levels of myo-inositol-1,4,5-triphosphate (182, 183). On the other hand, lithium can also reduce autophagy through GSK-3-beta inhibition which leads to activation of the mTOR pathway (184). Lithium may thus, through the mTOR pathway, to some degree decrease the translocation of TERT to mitochondria, potentially attenuating its beneficial effect of reducing the production of ROS. Conversely, combination treatment with lithium and rapamycin may have additive beneficial effects.

As defects in autophagy and the resulting lack of clearance of protein aggregates is considered to be an important contributing factor to neurodegeneration, the possibility of combining treatment with lithium and rapamycin in order to maximize autophagy through separate pathways have generated interest in the study of neurodegenerative disorders (166). Lithium and rapamycin have independently been found to have neuroprotective effects in animal models of Alzheimer’s disease, Parkinson’s disease, Huntington’s disease and fronto-temporal dementia (185–192). Moreover, in a study by Sarkar et al. (183), the authors found that combination treatment with lithium and rapamycin resulted in greater protection against neurodegeneration in a Huntington’s disease fly model compared to either treatment alone (183).

Interestingly, there are some indications that rapamycin may have beneficial effects in the treatment of mood disorders. In a study by Kara et al. (193), rapamycin was shown to reduce mania-like aggression and reward-seeking behaviors in the Black Swiss mouse-model of mania (193). Moreover, though the anti-depressive effect of ketamine has been suggested to be mediated by activation of mTOR based on pre-clinical studies (194), a recent randomized control trial found that mTOR inhibition increased the effect of antidepressants in patients with major depression (195). These findings highlight the potential benefits of using lithium and rapamycin in combination in treating various diseases of the central nervous system and should be further explored in the context of BD research.

## Conclusion

In this review, we have summarized current evidence of mitochondrial dysfunction, oxidative stress, and telomere shortening in BD and the effects of lithium on counteracting these cellular lesions. Moreover, we outlined the interdependent regulation of mitochondrial bioenergetics and telomere maintenance through ROS signaling and TERT. Furthermore, we proposed the hypothesis that transcriptional regulation of *TERT* and its subsequent nuclear and mitochondrial actions may explain part of lithium’s mood-stabilizing and neuroprotective effects. Finally, we have highlighted some recent studies on the beneficial neural effects of the inhibition of mTOR, which has also been shown to increase mitochondrial localization of TERT.

In order to clarify the potential role of TERT in lithium’s mechanism of action in BD, future studies should aim to (1) investigate the effect of lithium treatment on *TERT* gene expression (2) investigate the relative concentrations of TERT protein in the nucleus and mitochondria in response to oxidative stress, (3) investigate the different pathways that may mediate lithium’s effect on *TERT* expression and the effect of TERT on mitochondrial bioenergetics and oxidative stress, and (4) delineate the cellular effects of combining treatment with lithium and rapamycin. Moreover, we propose that investigating molecular differences between lithium responders and non-responders in this context will be of particular importance, as such findings could aid in the search for biomarkers of lithium response and new therapeutic targets in BD.

## Author Contributions

All authors contributed to the article and approved the submitted version. ML, VM, and MS wrote, while LB, LM, PS, CL, and CS edited the manuscript.

## Funding

This work was supported by a KID doctoral grant from Karolinska Institutet to MS, subsequently awarded to ML, the Swedish Research Council [MS, 2019-01651], the Swedish Brain Foundation [MS, FO2020-0309], the regional agreement on medical training and clinical research (ALF) between Stockholm County Council and Karolinska Institutet [MS, SLL2020-591461; LB, 2019-0237], and the Swedish Mental Health Fund [CL, 2019; LB, 2019].

## Conflict of Interest

PS serves on the scientific advisory board of Reata Pharmaceuticals Inc. CS is a scientific advisor to Outermost Therapeutics Inc. MS is on the scientific advisory board of NeuraMetrics Inc.

The remaining authors declare that the research was conducted in the absence of any commercial or financial relationships that could be construed as a potential conflict of interest.
